# Editorial: Experimental Hernia Research From Bench to Bedside and Translational Perspectives

**DOI:** 10.3389/fsurg.2021.695834

**Published:** 2021-06-17

**Authors:** Alexander H. Petter-Puchner, René H. Fortelny, Barbora East

**Affiliations:** ^1^Abdominal Wall Research, Institute for Experimental and Clinical Traumatology (LBG), Vienna, Austria; ^2^Faculty of Medicine, Visceral Surgery, Sigmund Freud University Vienna, Vienna, Austria; ^3^Second Faculty of Medicine, Charles University, Prague, Czechia

**Keywords:** translation, hernia research, bioengineering, technology, perspective

Hernia repair has been the pioneering field for innovative surgical techniques and devices. The past two decades have been a period of steady progress in order to reduce the incidence of chronic pain, raise the standards of elective surgery and develop feasible approaches for complex cases. The enormous number of procedures allows high quality clinical research and substantial support for experimental trials. This has led to constant influx of knowledge obtained in hernia research to other surgical domains, e.g., plastic and trauma surgery.

We are pleased to present this Research Topic, containing valuable contributions. These works reflect the continuous interest and striving activity of the research community and clinicians.

## Author Contributions

All authors listed have made a substantial, direct and intellectual contribution to the work, and approved it for publication.

## Conflict of Interest

The authors declare that the research was conducted in the absence of any commercial or financial relationships that could be construed as a potential conflict of interest.

